# Insights into the physiological role of CNS regeneration inhibitors

**DOI:** 10.3389/fnmol.2015.00023

**Published:** 2015-06-11

**Authors:** Katherine T. Baldwin, Roman J. Giger

**Affiliations:** ^1^Department of Cell and Developmental Biology, University of Michigan School of MedicineAnn Arbor, MI, USA; ^2^Cellular and Molecular Biology Graduate Program, University of Michigan School of MedicineAnn Arbor, MI, USA; ^3^Department of Neurology, University of Michigan School of MedicineAnn Arbor, MI, USA

**Keywords:** NogoA, MAG, OMgp, CSPGs, regeneration, synaptic plasticity

## Abstract

The growth inhibitory nature of injured adult mammalian central nervous system (CNS) tissue constitutes a major barrier to robust axonal outgrowth and functional recovery following trauma or disease. Prototypic CNS regeneration inhibitors are broadly expressed in the healthy and injured brain and spinal cord and include myelin-associated glycoprotein (MAG), the reticulon family member NogoA, oligodendrocyte myelin glycoprotein (OMgp), and chondroitin sulfate proteoglycans (CSPGs). These structurally diverse molecules strongly inhibit neurite outgrowth *in vitro*, and have been most extensively studied in the context of nervous system injury *in vivo*. The physiological role of CNS regeneration inhibitors in the naïve, or uninjured, CNS remains less well understood, but has received growing attention in recent years and is the focus of this review. CNS regeneration inhibitors regulate myelin development and axon stability, consolidate neuronal structure shaped by experience, and limit activity-dependent modification of synaptic strength. Altered function of CNS regeneration inhibitors is associated with neuropsychiatric disorders, suggesting crucial roles in brain development and health.

## Neural network plasticity—a delicate balancing act orchestrated by many players

Proper function of the adult mammalian CNS requires precise assembly, refinement, and maintenance of an elaborate network of neuronal connections. During development, axon guidance molecules facilitate the formation of intricate neuronal networks. After this initial assembly, many circuits are refined in an activity-dependent manner during a short time period of heightened plasticity, called the critical period (CP). At the end of the CP, the fine tuning of networks is complete and they acquire their mature shape. Synaptic contacts in the mature brain are stable over long time periods (Holtmaat and Svoboda, 2009), however, limited structural remodeling does occur and is thought to form the cellular basis of learning and the acquisition of new memories. Some degree of circuit plasticity is vital for proper brain function, and when improperly regulated can result in defects in learning and memory or cause nervous system diseases such as autism or schizophrenia (Mironova and Giger, 2013; Zagrebelsky and Korte, 2014).

To ensure rapid and accurate propagation of electrical impulses among different neural ensembles, most long axons are enwrapped with myelin sheaths. In addition to increasing the speed of impulse propagation, myelin provides metabolic support for axons (Franklin et al., 2012; Saab et al., 2013) and has neuroprotective properties (Franklin et al., 2012). Recent evidence shows that neuronal activity-regulated mechanisms exist that influence the extent of CNS myelination (Wake et al., 2011; Lundgaard et al., 2013; Gibson et al., 2014; Hines et al., 2015; Mensch et al., 2015), suggesting that adaptive myelination represents an as of yet underexplored form of activity-dependent nervous system plasticity. Indeed, perturbation of experience-dependent oligodendrocyte (OL) maturation may lead to neurological disorder (Long and Corfas, 2014).

Here we focus on the physiological function of CNS regeneration inhibitors in the naïve mammalian CNS. Vital roles for these molecules in the uninjured CNS raise important considerations for manipulating their function during therapeutic approaches directed toward augmenting neural plasticity and enabling nervous system repair.

## Synopsis of CNS regeneration inhibitors

### MAG

The neurite outgrowth inhibitory properties of MAG were discovered independently by the laboratories of Marie Filbin (Mukhopadhyay et al., 1994) and Peter Braun (McKerracher et al., 1994) more than 20 years ago. MAG is a type-1 transmembrane protein and a prominent member of the family of sialic acid-binding Ig superfamily (siglec) proteins. MAG is expressed by myelinating glia, Schwann cells in the periphery and oligodendrocytes (OL) in the CNS. MAG is abundant in the CNS and is enriched in Schmidt-Lanterman incisures and the periaxonal membrane of myelin sheath, allowing for complexes with receptors to form on the axonal surface (Trapp et al., 1989). The direct apposition of MAG and the axon membrane led to the early hypothesis that MAG plays an important role in regulating axon-myelin interactions and myelin development. *In vivo* studies with *Mag* knockout mice revealed surprisingly normal myelin development, yet closer examination uncovered a delay in OL differentiation and transient hypomyelination of the optic nerve in these mutants (Li et al., 1994; Montag et al., 1994; Pernet et al., 2008). At the ultrastructural level, peripheral and central nervous system myelin sheaths in *Mag* null mice display numerous subtle structural abnormalities, including aberrant myelin outfolds and uncompacted myelin wraps (Pernet et al., 2008). Additionally, loss of MAG delays node of Ranvier formation and alters distribution of nodal proteins, including paranodin and sodium channels (Marcus et al., 2002). Aging studies in *Mag* knockout mice revealed increased axonal “drop out” and axonal atrophy, indicating that MAG plays a crucial role in maintenance and long-term stability of the axon-glial unit (Fruttiger et al., 1995).

Our understanding of the molecular mechanisms employed by MAG to exert its different functions is still incomplete. Several receptors for MAG have been identified, including the complex brain gangliosides GD1a and GT1b (Yang et al., 1996), the Nogo receptor family members NgR1 (Domeniconi et al., 2002; Liu et al., 2002) and NgR2 (Venkatesh et al., 2005), paired Ig-like receptor B (PirB) (Atwal et al., 2008), β1-integrin (Goh et al., 2008), and low density lipoprotein receptor-related protein 1 (LRP1). Except for the interaction with LRP1, MAG binds to its neuronal surface receptors in a sialic acid-dependent manner (Robak et al., 2009; Stiles et al., 2013). Myelination and nodal defects in *B4galnt1* null mice, which lack major brain gangliosides (including GD1a and GT1b), display striking similarities to *Mag* null mice, suggesting that gangliosides may be the primary receptors responsible for MAG-mediated axon protection (Schnaar and Lopez, 2009). Whether NgR1, NgR2, PirB, β1-integrin, or LRP1 play a role in MAG mediated axon protection *in vivo* has not yet been addressed conclusively and will likely require the generation of compound mutant mice to deal with potential redundant functions among different MAG receptors. MAG modulates the axonal cytoskeleton, affecting axon caliber, neurofilament spacing (Yin et al., 1998), post-translational modification of microtubules (Hsieh et al., 1994; Nguyen et al., 2009), phosphorylation of MAP2 (Dashiell et al., 2002) and activation of the RhoA/ROCK signaling pathway (Mimura et al., 2006). In future studies it will be important to determine whether the molecular mechanisms of MAG-mediated axon protection and neurite outgrowth inhibition can be dissociated, and if so, whether this can be exploited therapeutically to selectivity promote axon protection while at the same time eliminating neuronal growth inhibitory constraints imposed by MAG.

### NogoA

NogoA is a membrane-associated protein that belongs to the reticulon family (GrandPre et al., 2000). Originally identified as a neurite growth inhibitory “activity” enriched in a spinal cord white matter fraction (Caroni and Schwab, 1988; Caroni et al., 1988), three laboratories described the molecular identity of Nogo-A more than 15 years ago (Chen et al., 2000; GrandPre et al., 2000; Prinjha et al., 2000). NogoA harbors at least two distinct growth inhibitory motifs, Nogo-66 (Fournier et al., 2001) and NogoΔ20 (Oertle et al., 2003). In the injured spinal cord, acute antibody blockade of Nogo-A promotes axon sprouting and is associated with improved behavioral outcomes (Schnell and Schwab, 1990; Merkler et al., 2001; Liebscher et al., 2005). NogoA is expressed by many cell types, though its expression is highest in OLs and principal neurons in brain regions with a heightened degree of network plasticity, including the hippocampus and neocortex (Huber et al., 2002; Zhang et al., 2014). In the OL lineage, NogoA appears to be an important regulator of myelin development. Treating oligodendrocyte precursor cells (OPCs) with a Nogo-A function-blocking antibody impairs differentiation of OPCs into mature OLs *in vitro* (Huang et al., 2012). Additionally, *NogoA* knockout mice show reduced OPC differentiation *in vivo* (Chong et al., 2012). Similar to *Mag* null mice, the optic nerves in *NogoA* null mice are hypomeylinated during development, but not in adulthood (Pernet et al., 2008). NogoA also participates in contact-mediated competitive interactions between OLs to regulate the myelogenic potential (Chong et al., 2012). Cocultures of WT and *NogoA* null OPCs revealed that the spacing of myelin internodes formed by WT OLs depends on NogoA expression in neighboring OLs. This regulation is likely accomplished by NogoΔ20, since bead-bound NogoΔ20 significantly inhibits the number of myelin internodes formed per OL in culture. *In vivo*, *NogoA* null mice show expansive and aberrant myelination, including hypermyelination of the superficial layers in the neocortex (Chong et al., 2012).

### OMgp

OMgp is a 110-kDa leucine-rich repeat protein linked to the cell membrane by a glycosylphosphatidylinositol (GPI)-anchor. OMgp is expressed by OLs and neurons in the CNS (Vourc'h et al., 2003) and is also found in astrocytes (Zhang et al., 2014). Two independent studies identified OMgp as a potent growth inhibitory molecule enriched in CNS myelin (Kottis et al., 2002; Wang et al., 2002). Compared to MAG and NogoA, significantly less is known about the physiological role of OMgp. However, there may be some degree of overlap, as OMgp, similar to NogoA and MAG, associates with NgR1 and PirB. Analogous to NogoA, antibody blockade of OMgp leads to impaired differentiation of OPCs into mature OLs *in vitro* (Huang et al., 2012). *OMgp* null mice display defects of the nodal and paranodal architecture (Nie et al., 2006) and hypomyelination of the spinal cord that correlates with slower propagation of ascending and descending electrical impulses (Lee et al., 2011). OMgp was shown to be enriched near nodes of Ranvier, where it reportedly blocks axon collateral sprouting from non-myelinated segments (Huang et al., 2005). However, a subsequent study, using the same anti-OMgp antiserum in WT and *OMgp* knockout tissue, showed that *OMgp* is not enriched at nodes (Chang et al., 2010). Additional studies are needed to describe the physiological properties of OMgp *in vivo* and to define the extent to which this underexplored molecule contributes to aspects of axon-myelin communication.

### CSPGs

Another prominent group of CNS regeneration inhibitors, chondroitin sulfate proteoglycans (CSPGs), are extracellular matrix (ECM) proteoglycans that consist of a protein core with covalently attached glycosaminoglycan (GAG) side chains (Properzi et al., 2003; Silver and Silver, 2014). CSPGs are secreted by astrocytes, neurons, and oligodendrocytes (Ogawa et al., 2001), and they are strongly enriched at the glial scar after CNS injury where they inhibit regenerative growth and restrict plasticity (Bradbury et al., 2002; Morgenstern et al., 2002; Silver and Miller, 2004). CSPGs are a major component of the brain ECM with developmentally regulated expression, therefore they are thought to play a role in neural development, axon guidance, and synaptic plasticity (Kwok et al., 2012). During development, immature OLs express brevican precisely when they extend processes to ensheathe axons (Ogawa et al., 2001), and loss of brevican perturbs ECM distribution near nodes of Ranvier in adulthood (Bekku et al., 2009). As discussed below, CSPGs play important roles in visual system development and plasticity (Pizzorusso et al., 2002), and also in the formation, refinement, and modification of synaptic structures in other brain regions (Orlando et al., 2012) and the protection of memories from erasure (Gogolla et al., 2009).

## CNS regeneration inhibitors consolidate neuronal architecture at the end of critical periods

Proper formation, maintenance, and activity-dependent modification of synaptic contacts may be achieved through dynamic regulation of molecules that promote structural plasticity and also molecules that stabilize existing structures (Mironova and Giger, 2013; de Wit and Ghosh, 2014; Zagrebelsky and Korte, 2014). Strong evidence that CNS regeneration inhibitors may be involved in limiting neuronal plasticity stems from work in the rodent visual system. In the juvenile brain, normal development of the primary visual cortex involves a CP of heightened plasticity. During the CP, visual experience drives refinement of visual neuronal architecture, including the formation of ocular dominance (OD) columns. Once the CP is closed, mature networks are maintained and OD plasticity is more restricted (Levelt and Hubener, 2012). Though most extensively studied in the visual cortex, activity-dependent refinement of neural circuits is not unique to the visual system. CPs exist in many other brain regions and are important for the acquisition of language and certain forms of higher cognitive processing. Elegant studies by Pizzorusso et al. showed that enzymatic digestion of CSPG glycosaminoglycan chains by local infusion of chondroitinaseABC, a bacterial enzyme that digests the GAG side chains on CSPGs, greatly augments OD plasticity in the binocular zone of the adult visual cortex (Pizzorusso et al., 2002). McGee and colleagues found that in *NgR1* mutant mice, there is no temporal limit to the CP, and robust OD plasticity persists throughout adulthood (McGee et al., 2005). In a similar vein, OD plasticity is extended in *Nogo* (McGee et al., 2005) and *PirB* (Syken et al., 2006) mutant mice, indicating that CNS regeneration inhibitors and their receptors function as negative regulators of experience-driven neuronal plasticity at the end of the CP in the visual system. Thus, one important physiological function of CNS regeneration inhibitors is to consolidate neuronal architecture established at the end of CPs, thereby stabilizing microcircuits that are highly tuned and difficult to assemble.

### Neuronal expression of inhibitory ligands and their receptors

CNS regeneration inhibitors are expressed by glia and neurons. Nogo-A, OMgp and several CSPGs are expressed by neurons and found along axons and dendrites (Mironova and Giger, 2013). Interestingly, Nogo-A and OMgp are present in presynaptic and post-synaptic density fractions isolated from the rodent hippocampus (Lee et al., 2008; Raiker et al., 2010). In a similar vein, NgR1 a receptor for Nogo-66, OMgp and CSPGs, is also found in synaptic density fractions, as is PirB (Fournier et al., 2001; Wang et al., 2002; Atwal et al., 2008; Filbin, 2008). PirB is a member of the leukocyte immunoglobulin-like receptor family (LILRB3), and compared to NgR1 it is much less prominently expressed in the CNS (Raiker et al., 2010; Zhang et al., 2014). The NogoΔ20 domain of NogoA does not interact with PirB or members of the Nogo receptor family. A recent study identified sphingosine 1 phosphate receptor 2 (S1PR2) as a novel receptor for NogoΔ20 (Kempf et al., 2014). S1PR2 is expressed in hippocampal neurons, and as discussed below, it is necessary for NogoΔ20-elicited changes in synaptic function (Nie et al., 2006). CSPGs are secreted by astrocytes and neurons, and they are found at synaptic sites and enriched in perineuronal nets (PNNs) (Orlando et al., 2012; Miao et al., 2014). A number of neuronal surface receptors bind CSPGs and inhibit neurite outgrowth *in vitro*, including NgR1, NgR3, leukocyte common antigen-related protein (LAR), and its homolog RPTPσ (Shen et al., 2009; Fisher et al., 2011; Dickendesher et al., 2012). NgR1, LAR, and RPTPσ are present both pre and post-synaptically (Mironova and Giger, 2013; Takahashi and Craig, 2013). Challenges for future studies, therefore, will be to dissect which CSPGs exert their functions through which receptor complexes and to define their functional relationship to different classes of ligands already known to operate in a LAR family or Nogo receptor family dependent manner.

### CNS regeneration inhibitors can influence synaptic structure and density

Dendritic spines are specialized post-synaptic compartments that receive excitatory inputs from presynaptic axon terminals. Dendritic spines display various morphologies, ranging from immature thin or filopodia-like protrusions to mature mushroom-shaped structures. Spine morphology is thought to reflect the maturity and strength of excitatory synaptic connections (Sala and Segal, 2014). In the hippocampus of *NgR1* knockout mice, CA1 dendritic spines have a less mature distribution profile than wild-type littermate controls, suggesting that NgR1 is required for the proper development or maintenance of mature spines (Lee et al., 2008). Studies with Nogo receptor compound mutant mice revealed that loss of all three NgR family members (NgR1, NgR2, and NgR3) increases synaptic density in the juvenile hippocampus, suggesting that NgRs function as negative regulators of synaptogenesis. In primary hippocampal neurons, loss of any one NgR family member increases dendritic spine density. *In vivo*, however, an increase in spine density is only observed in *NgR* triple knockout mice, suggesting some degree of functional redundancy among these related molecules (Wills et al., 2012). In a recent study NgR1 was reported to be a key molecule in limiting dendritic spine turnover in the somatosensory cortex of adult mice (Akbik et al., 2013). However, this finding was challenged by a subsequent study that found no role for NgR1 in restricting dendritic spine turnover using the same transgenic mouse model (Park et al., 2014). It is unclear which NgR ligands are responsible for regulation of synaptic density and dendritic spine morphology, though CSPGs, NogoA, and OMgp are present in synaptic density fractions and therefore are likely candidates (Raiker et al., 2010; Takahashi and Craig, 2013). Similar to NgR1, NogoA promotes spine maturation in hippocampal pyramidal neurons. In hippocampal slice cultures, antibody blockade of NogoA shifts dendritic spine morphology toward a more immature phenotype but does not affect spine density (Zagrebelsky et al., 2010). *In vivo*, administration of NogoA function-blocking antibodies into the motor cortex of adult male rats leads to a net increase in dendritic spine density due to an increase in spine formation (Zemmar et al., 2014).

The correlation of ECM maturation with reduced spine dynamics at the closure of the CP suggests that CSPGs, since they are ECM components, may play an active role in restricting spine formation and maturation. Indeed, treatment of hippocampal slices with ChaseABC to digest perisynaptic CSPGs increases dendritic spine density and formation of spinehead protrusions through a mechanism that requires β1-integrin (Orlando et al., 2012). In neuron-glia co-cultures, enzymatic digestion of brevican and neurocan GAG chains promotes formation of synaptic puncta (Pyka et al., 2011), suggesting that CSPGs restrict synaptogenesis. CSPGs may restrict synapse formation through a mechanism involving RPTPσ or NgRs since mice lacking these receptors display increased dendritic spine density and length *in vivo* (Horn et al., 2012; Wills et al., 2012).

### Regulation of synaptic function

In the mature brain, alterations in neuronal structure are thought to reflect prolonged changes in neuronal activity. Long-term potentiation (LTP) and long-term depression (LTD) of synaptic transmission are opposing forms of activity-dependent synaptic strength modifications, and are thought to underlie aspects of learning and memory formation (Siegelbaum and Kandel, 1991). Neurotrophic factors such as fibroblast growth factors (FGFs) promote neuronal growth and plasticity and may antagonize the growth inhibitory effects of CNS regeneration inhibitors. In acutely isolated hippocampal slices of WT mice, exogenously applied FGF2 does not alter LTP at CA3-CA1 synapses. In *N*gR1^−/−^ slices, LTP at CA3-CA1 synapses is indistinguishable from WT controls. However, exogenous application of FGF2 greatly increases LTP in *N*gR1^−/−^ slices. Pharmacological studies show that elevated LTP in *N*gR1^−/−^ slices requires FGFR kinase activity (Lee et al., 2008). In juvenile hippocampal slices of *N*gR1^−/−^ mice, NMDAR dependent LTD at CA3-CA1 synapses was absent, suggesting that synaptic depression requires NgR1 signaling (Lee et al., 2008). Moreover, treatment of acute hippocampal slices with soluble Nogo66 or OMgp suppresses hippocampal LTP in an *NgR1*-dependent manner (Raiker et al., 2010). These results demonstrate that CNS regeneration inhibitors and their receptors can regulate the strength of synaptic transmission, and may accomplish this, in part, by antagonizing growth promoting signaling pathways.

NogoA strongly attenuates LTP at hippocampal CA3-CA1 synapses, since it is observed that antibody blockade of the NogoΔ20 region leads to increased LTP (Delekate et al., 2011). Counter-intuitively, treatment of hippocampal slices with soluble NogoΔ20 leads to an increase in LTP. This NogoΔ20-mediated increase in LTP may be caused by rapid endocytosis of NogoA (Joset et al., 2010), resulting in an opposite effect since internalization of the receptor complex attenuates NogoΔ20 signaling. Surprisingly, recordings from acute hippocampal slices of *NogoA* knockout mice show normal LTP and normal LTD at CA3-CA1 synapses, suggesting that related mechanisms exist that compensate for chronic loss of NogoA (Delekate et al., 2011). Inhibition of the NogoΔ20 receptor S1PR2 enhances LTP in the hippocampus and motor cortex of WT mice, but not in *NogoA* knock-out mice (Kempf et al., 2014), indicating that NogoA may restrict activity-dependent plasticity through S1PR2. Collectively, these studies establish the NogoΔ20 region of NogoA as a negative regulator of activity-dependent synaptic plasticity.

CSPGs are known to influence activity-dependent synaptic strength, but the mechanisms are less clear. Mice lacking *RPTPσ* display altered basal synaptic transmission, including greater paired-pulse facilitation along with increased frequency of miniature excitatory post-synaptic currents (mEPSCs) (Horn et al., 2012). Additionally, *RPTPσ* null mice have reduced LTP, yet enhanced novel object recognition memory (Horn et al., 2012). These alterations in synaptic function may not be specific to CSPGs, since RPTPσ, similar to NgR1 and NgR3, also interacts with heparan sulfate proteoglycans (HSPGs) (Coles et al., 2011; Dickendesher et al., 2012). The cross-talk between CSPG and HSPG family members at the synapse, and in neural network plasticity in general, is an exciting but underexplored issue.

### Signaling pathways

Collectively, the above studies suggest that an intricate cross-talk occurs between growth promoting and growth inhibitory signaling pathways to achieve proper regulation of synaptic plasticity and stability. Cross-talk between CNS regeneration inhibitors and growth factors that increase plasticity may occur at multiple levels. This is well illustrated by examining how CNS regeneration inhibitors antagonize brain-derived neurotrophic factor (BDNF) signaling. At the cell surface, BDNF binds TrkB to activate several growth promoting pathways, including mTOR complex 1 (mTORC1) and MAP kinase/ERK. However, CSPG binding to RPTPσ attenuates activity of TrkB (Kurihara and Yamashita, 2012). At the level of cell signaling and protein translation, BNDF-mediated activation of mTORC1 leads to increased local protein synthesis at dendritic spines and promotes synaptic plasticity (Tang et al., 2002; Leal et al., 2014). In primary cortical neurons, pretreatment with crude CNS myelin or recombinant Nogo66 attenuates the BDNF-mediated increase in phosphorylation of p70S6K, a downstream target of mTORC1 (Raiker et al., 2010). This suggests that NogoA and NgR1 may restrict synaptic plasticity through negative regulation of mTORC1 signaling, and perhaps the (local) translation of synaptic proteins. In synaptic density fractions isolated from the hippocampus of *NgR1* mutant mice, levels of phosphorylated ERK are significantly increased, suggesting that NgR1 negatively regulates ERK signaling (Lee et al., 2008). Cross-talk might also occur at the transcriptional level through regulation of cAMP response element-binding protein (CREB). Elevating cAMP levels overcomes myelin-mediated inhibition of neurite outgrowth in a CREB dependent manner (Gao et al., 2004), while NogoΔ20 decreases activation of CREB (Joset et al., 2010).

## Role of CNS inhibitory molecules in CNS disorders

Many human neuropsychiatric disorders are associated with defects in synaptic structure or function and may be caused by a shift in excitatory/inhibitory balance (Pittenger, 2013). Given that CNS myelin inhibitors play important roles in regulating these processes, altered expression or function may contribute to developmental brain disorders. In the aging brain, hippocampal expression of several CNS regeneration inhibitors increases, and correlates with, deficits in spatial learning and memory (Vanguilder et al., 2012). *NgR1* null mice show impaired memory function, including impaired fear extinction and consolidation (Park et al., 2014) as well as slow acquisition of a spatial memory task (van Gaalen et al., 2012). Rats with reduced expression of NogoA display defects in cognition and social behavior associated with schizophrenia (Tews et al., 2013). Interestingly, mutations in both human *NgR1* and *NogoA* have been associated with schizophrenia (Sinibaldi et al., 2004; Willi and Schwab, 2013).

## Concluding remarks

CNS regeneration inhibitors play important physiological roles in the uninjured brain and spinal cord. The myelin-associated inhibitors MAG, NogoA, and OMgp regulate myelin formation and axon-myelin interactions. NogoA, OMgp, and CSPGs regulate synapse formation and maturation, and they influence activity-dependent synaptic strength. Growing evidence suggests that some CNS regeneration inhibitors participate in an intricate cross-talk with growth promoting molecules at the level of several key signaling molecules. This finely tuned balance of excitation and inhibition in the developing and mature CNS may be necessary for proper formation and function of neural networks.

The physiological role of these molecules raises important considerations for therapeutic strategies designed to promote neural regeneration following injury. The acquisition of a large number of ligand-receptor systems that restrict neural network plasticity may have been a prerequisite that enabled the evolution of larger and more powerful neural networks. Larger and more complex brains may be more vulnerable to aberrant changes in synaptic connectivity and therefore, once fully developed, need to be consolidated and protected by molecules that constrain excessive network rearrangement. Following injury to the adult CNS, molecules that restrict aberrant growth and plasticity may be detrimental since they limit attempts to modify or rebuild nearby networks to compensate for lost neural circuits. Therefore, a deeper understanding of the physiological roles played by CNS regeneration inhibitors is of great interest both clinically and biologically.

### Conflict of interest statement

The authors declare that the research was conducted in the absence of any commercial or financial relationships that could be construed as a potential conflict of interest.
